# A paradigm shift in trichinellosis management: curcumin-olive oil nanocomposite’s multi-faceted therapeutic approach

**DOI:** 10.1186/s12917-025-04821-w

**Published:** 2025-05-22

**Authors:** Reem M. Ramadan, Marwa M. Khalifa, Fady Sayed Youssef, Ehab A. Fouad, Mohamed Kamel, Mohamed M. El-Bahy, Noha Madbouly Taha

**Affiliations:** 1 Department of Parasitology, Faculty of Veterinary Medicine, Cairo University, Giza, 12211 Egypt; 2 Department of Pharmacology, Faculty of Veterinary Medicine, Cairo University, Giza, 12211 Egypt; 3 Department of Zoonosis, Veterinary Research Institute, National Research Centre, Giza, Egypt; 4 Department of Medicine and Infectious Diseases, Faculty of Veterinary Medicine, Cairo University, Giza, 11221 Egypt; 5 Department of Parasitology, Faculty of Medicine, Cairo University, Giza, Egypt

**Keywords:** *Trichinella spiralis*, Treatment, Curcumin-olive oil nanocomposite, Redox parameters, Oxidative stress

## Abstract

**Background:**

*Trichinella spiralis*, a globally widespread zoonotic parasite, poses significant health and economic burdens due to its complex life cycle and the scarcity of effective, multi-stage treatments.

**Methods:**

This study investigated the therapeutic potential of a novel curcumin-olive oil nanocomposite (CO-NC) against three critical stages of *T. spiralis* infection in a murine model: adult worms (3–5 days post-inoculation, dpi), newborn larvae (8–10 dpi), and encapsulated larvae (33–35 dpi). CO-NC exhibited potent, stage-specific, and dose-dependent antiparasitic activity.

**Results:**

Remarkably, a 100 mg/kg dose achieved complete eradication of both newborn and encapsulated larvae, mirroring the efficacy of the current standard treatment, albendazole (50 mg/kg). This high dose also significantly reduced adult worm burdens by 91.6%. Even at a lower dose of 50 mg/kg, CO-NC demonstrated substantial activity, reducing adult worms and encapsulated larvae by 55.2% and 43.8%, respectively. Beyond its direct antiparasitic effects, CO-NC (100 mg/kg) significantly mitigated infection-induced oxidative stress by restoring key redox markers in muscle and intestinal tissues, including xanthine oxidase, glutathione, malondialdehyde, and total antioxidant capacity. Furthermore, complementary in vitro studies revealed superior anticancer and anti-inflammatory properties of CO-NC compared to crude curcumin and standard reference compounds at their respective IC_50_ values.

**Conclusions:**

These findings highlight CO-NC as a promising multi-faceted therapeutic candidate for trichinellosis, offering potent antiparasitic efficacy comparable to albendazole alongside valuable antioxidant, anti-inflammatory, and anticancer properties. This integrated approach underscores the potential of CO-NC as an innovative and comprehensive solution for the challenges posed by *T. spiralis* infections.

## Introduction

*Trichinella spiralis* (*T. spiralis*), a globally distributed zoonotic nematode, is the etiological agent of trichinellosis, a parasitic disease that poses significant public health and economic burdens worldwide. This parasite exhibits a complex life cycle, infecting the intestines and muscles of a diverse range of hosts, including mammals, birds, and reptiles [1]. Human infection primarily occurs through the consumption of undercooked or raw meat harboring infective larvae. Upon ingestion, these larvae mature into adults within the intestinal mucosa, subsequently producing newborn larvae approximately 5–7 days post-infection (dpi). These newborn larvae then migrate via the bloodstream to striated muscle fibers, particularly in the diaphragm and tongue, where they encapsulate and remain infective [2]. Notably, undercooked pork, bear, and horse meat are the predominant sources of human trichinellosis.

Current therapeutic interventions for trichinellosis demonstrate limited efficacy, especially against the encapsulated larval stage, which is notably resilient. Commercially available anthelmintics often exhibit suboptimal performance across various parasitic stages, primarily due to their poor water solubility and low bioavailability [3]. These limitations underscore the urgent need for novel, efficacious anthelmintic therapies [4]. In this context, plant-derived bioactive compounds have emerged as promising alternatives, offering potential therapeutic efficacy coupled with a more favorable safety profile. Consequently, there is growing interest in investigating the antiparasitic potential of medicinal plants and herbal remedies traditionally used for therapeutic purposes [5].

Curcumin, a polyphenolic compound derived from the rhizome of *Curcuma longa*, has been extensively studied for its diverse pharmacological properties. This bioactive molecule exhibits a broad spectrum of activities, including anti-inflammatory, anticancer, antimicrobial, gastroprotective, neuroprotective, and wound-healing effects [6]. Moreover, curcumin has demonstrated significant antiparasitic efficacy against a wide range of pathogens, including species of *Leishmania*, *Plasmodium*, *Entamoeba*, Trichomonas, *Giardia*,

*Toxoplasma*, *Cryptosporidium*, *Eimeria*, *Schistosoma*, and *Fasciola* [7]. However, the therapeutic potential of curcumin is hampered by its poor water solubility, low bioavailability, and rapid degradation during preparation and delivery. To address these limitations, nanotechnology-based formulations, such as nanosized curcumin (particles measuring 1–100 nm), have been developed, significantly enhancing its stability, solubility, and efficacy in medical applications [3].

The pathogenesis of trichinellosis is characterized by significant tissue damage, which is closely associated with inflammatory responses and oxidative stress. This is evidenced by elevated levels of oxidative stress markers, including xanthine oxidase (XO), glutathione-S-transferase omega-1 (GSTO-1), and malondialdehyde (MDA), coupled with depleted glutathione (GSH) levels [8]. Consequently, antioxidant therapies have emerged as a promising approach to mitigate these pathological effects by attenuating oxidative stress and inflammation [9].

Recently, a novel curcumin-olive oil nanocomposite (CO-NC) was developed to overcome the limitations associated with conventional curcumin formulations. This innovative nanocomposite is characterized by enhanced stability, improved bioavailability, and nanoscale dimensions [10]. Our previous in vitro studies have demonstrated significant antiparasitic activity of CO-NC, with trichinocidal effects observed at concentrations as low as 40 ppm over a 12-hour period [11]. Furthermore, CO-NC has exhibited potent activity against oocysts of *Eimeria* species, underscoring its potential as a broad-spectrum antiparasitic agent.

Building on recent advances, nanotechnology has emerged as a promising strategy to enhance drug delivery. Despite this progress, several key challenges remain. Chief among these are the propensity of nanoparticles to self-aggregate at low concentrations of loaded material and the issue of polydispersity, both of which can compromise product stability and lead to variability in drug entrapment. Particle size is another critical factor; while particles smaller than 20 nm are preferred for optimal loading, those exceeding 100 nm often display reduced stability and bioavailability. Addressing these limitations is essential for realizing the full potential of nanotechnology-based drug delivery systems [12].

In light of these challenges and the promising findings, the present study aims to comprehensively evaluate the therapeutic potential of CO-NC against *T. spiralis* infection. Specifically, we assess the safety profile, cytotoxicity, antioxidant capacity, anti-inflammatory properties, and anticancer potential of CO-NC in comparison to crude curcumin through in vitro assays. Additionally, we investigate the in vivo efficacy of CO-NC as a trichinocidal agent against multiple life stages of *T. spiralis*, including adult worms, migrating newborn larvae, and encapsulated larvae, in experimentally infected mice. Finally, we explore the capacity of CO-NC to mitigate infection-induced oxidative stress by monitoring key redox parameters in treated and control animals.

## Methods

### Ethical approval and source of mice

Mice used in this study were purchased from a commercial animal breeding facility (Vacsera, Egypt). Upon arrival, the animals were housed in a controlled environment under standardized conditions for a seven-day acclimatization period prior to the commencement of the experiments. All experimental protocols were approved by the Institutional Animal Care and Use Ethical Committee of Cairo University (Vet CU 01122022623), and all methods were carried out in accordance with relevant guidelines and regulations. Since the animals were sourced from a university breeding unit, no additional owner consent was required.

### Production of curcumin-olive oil nanocomposite (CO-NC)

#### Materials used

To produce the CO-NC, three key components were utilized: curcumin powder in its pure form (Sigma-Aldrich Company), Tween 80 as a surfactant (Al-Nasr Pharmaceuticals, Cairo, Egypt), and olive oil (Sekem Company, Cairo, Egypt). These materials were acquired under strict quality control and used immediately to ensure the integrity of the product during preparation.

#### Preparation of CO-NC

The synthesis of curcumin nanoparticles was carried out using a sonochemical method, as described by Alsuraihi et al. 2023 [13]. In this process, Tween 80 was employed to create a stable oil phase, enabling the dispersion of curcumin within a water-based medium. The resulting nanocomposite was formulated into a water-soluble solution, with a concentration of 100 ppm of active curcumin per milliliter. This preparation method ensured consistent particle formation and stability.

#### Characterization of CO-NC

To comprehensively understand the physical properties of the CO-NC, advanced analytical techniques were employed. Particle size and distribution were analyzed using dynamic light scattering (DLS) with the NanoSight NS500 model (Malvern Instruments Ltd, UK). Additionally, the morphological features of the nanoparticles were examined using scanning electron microscopy (SEM) with a Prisma E (Thermo Scientific Company) instrument and transmission electron microscopy (TEM) using an EM-2100 high-resolution magnification device. These methods provided a detailed characterization of the shape and size of the nanoparticles, ensuring their suitability for downstream applications.

#### Determination of LD50

The median lethal dose (LD50) of the CO-NC was determined to assess its safety profile, following the protocol described by Hamed et al. 2022 [14]. Swiss albino mice (20–25 g) were obtained from the Laboratory Animal Breeding Unit, Department of Animal and Poultry Management and Behaviour, Faculty of Veterinary Medicine, Cairo University [15]. Five groups of mice (*n* = 5 per group) were orally administered CO-NC at graded doses of 1,000, 3,000, 5,000, 8,000, and 10,000 mg/kg body weight (bw), while a sixth group served as a non-treated control. The mice were observed for three consecutive days post-administration to monitor mortality and assess hepatic and renal function, as well as histopathological changes such as tissue distortion, inflammatory cell infiltration, and necrotic signs.

The LD50 value was calculated using the following formula:

LD50 = DM - (∑AXB) / NWhere:

- DM = the highest dose killing all mice.
- A = average number of dead mice between two successive doses.
- B = interval between two successive doses.
- N = number of mice per group.
- Σ = (A × B).

This calculation provided a reliable estimate of the dose at which 50% of the mice population succumbed, thus helping establish the safety threshold for CO-NC [16].

### Biological characterization of CO-NC

#### Evaluation of antioxidant activity

To explore the antioxidant properties of CO-NC, the 2,2-diphenyl-1-picrylhydrazyl (DPPH) radical scavenging assay was employed, as outlined by Hamed et al. (2022) [14]. A series of dilutions (ranging from 0.19 to 100 ppm as 100-50-25-12.5-6.25-3.12-1.56-0.78-0.39 and 0.19 ppm) were prepared by diluting the CO-NC stock solution with distilled water. Each dilution (1 mL) was then mixed with 1 mL of DPPH solution (1 mM in methanol) and incubated in the dark at room temperature for 30 min. After the incubation, absorbance was measured at 517 nm using a UV-Vis spectrophotometer (Systronics AU-2701).

Ascorbic acid (Vitamin C) was used as a positive control for antioxidant activity. The scavenging activity of CO-NC was calculated using the following formula:

%ScaV = (Pc– Ps) / Pc X 100 Where:

- Pc = absorbance of the DPPH control.
- Ps = absorbance of the CO-NC or Vitamin C sample.

This method provided a quantitative assessment of the ability of CO-NC to neutralize free radicals, highlighting its potential as an effective antioxidant.

#### Cytotoxicity assay on HepG2 cells

The cytotoxic effects of CO-NC were evaluated against the HepG2 liver cancer cell line (ATCC: HB-8056) using the Methyl-Thiazolyl Tetrazolium (MTT) assay. HepG2 cells were cultured to form a monolayer and treated with serial dilutions of CO-NC and crude curcumin. After 24 h, MTT reagent (Bio Basic Canada Inc.) was added to the wells, followed by incubation at 37 °C in a humidified atmosphere with 5% CO2. The resulting formazan crystals were dissolved, and absorbance was measured at 560 nm, with background subtraction at 620 nm.

The concentration of CO-NC required to inhibit 50% of cell viability (IC50) was calculated from the dose-response curve, following the methodology of Kandeel et al. (2022) [17]. This assay provided insight into the potential therapeutic effects and safety of CO-NC.

#### Anti-inflammatory activity evaluation

The anti-inflammatory potential of CO-NC was tested by evaluating its ability to inhibit cyclooxygenase enzymes (COX-1 and COX-2) using a COX inhibitor screening assay kit (Cayman Chemicals, 560131, MI, USA). The assay was conducted according to the protocols described by Kazemzadeh et al. 2014 and Moryani et al. 2021 [18, 19]. This provided a detailed understanding of the capacity of CO-NC to modulate inflammatory pathways.

### In vivo trichinocidal efficacy of CO-NC

#### T. spiralis infection model

Swiss albino mice (6–8 weeks old, 28–32 g) were housed under standard laboratory conditions, with ad libitum access to water and balanced rodent feed. *T. spiralis* larvae were extracted from naturally infected pig diaphragms using a digestion method (1% pepsin-HCl), as described by Mayer-Scholl et al. 2017 [20]. The larvae were counted using a McMaster chamber and confirmed morphologically and genetically via PCR amplification of the COXI locus (GenBank accession number: OR271983) [21, 22]. Mice were orally inoculated with 200 larvae/mouse [23].

#### Treatment regimen and administration

Mice were divided into three groups (G1, G2, G3) based on the parasite stage:

- Adult worms (G1): Treated on days 3, 4, and 5 post-infection (dpi).
- Newborn larvae (G2): Treated on days 8, 9, and 10 dpi.
- Encysted larvae (G3): Treated on days 33, 34, and 35 dpi.

Each group was further subdivided into four subgroups (*n* = 7 each) receiving 50 mg/kg or 100 mg/kg CO-NC, albendazole (ABZ) (50 mg/kg, reference drug), or no treatment (control). Treatments were administered orally using an esophageal tube. Albendazole 2.5% solution (produced by Pharma Swede) was used as the reference anti-helminthic drug at a dose of 50 mg/kg B.W. orally [24, 25].

#### Efficacy evaluation metrics

Three days post-treatment, mice were euthanized using isoflurane (5% in oxygen; Ohmeda Isotec 4 Vaporizer), and their intestinal and muscle tissues were processed to recover worms and larvae using digestion and sedimentation techniques [26]. Drug efficacy was calculated using:

$${\rm{ Cutworm/larvae}}\;{\rm{rate}}\; = \;{\matrix{{\rm{Mean }}\;{\rm{number}}\;{\rm{from}}\;{\rm{the}}\;{\rm{control}}\; - \; \hfill \cr {\rm{Mean}}\;{\rm{number}}\;{\rm{from}}\;{\rm{the}} \hfill \cr \;{\rm{drug}}\;{\rm{inoculated groups}} \hfill \cr} \over \matrix{{\rm{Mean}}\;{\rm{number}}\;{\rm{from}}\; \hfill \cr {\rm{the}}\;{\rm{control}} \hfill \cr} } \times 100$$

#### Redox parameters analysis

Biochemical analyses of intestinal and muscle tissues were performed to measure redox biomarkers, including TAC, MDA [27], GSH [28], and XO activity, using commercially available kits [29].

### Statistical analysis

Statistical analyses were performed using SPSS software version 27 [30]. One-way ANOVA with Fisher’s LSD post-hoc test was used for single-factor comparisons, while two-way ANOVA with Fisher’s LSD post-hoc test was applied for two-factor analyses (e.g., treatment types and concentrations) [31]. A significance level of α = 0.05 was used for all tests [32].

## Results

The experimental workflow detailing the evaluation of the biological characterization and therapeutic efficacy of CO-NC was presented. Specifically, the study systematically investigated its safety profile, antioxidant activity, cytotoxicity, anti-inflammatory effects, and in vivo efficacy against *T. spiralis*. By combining these findings, the experiments provide a thorough assessment of CO-NC’s potential as a multifunctional therapeutic agent.

### Characterization

The average particle size of CO-NC was found to be 63 nm with a polydispersity index (PDI) of 0.41, ideal for distribution. SEM & TEM images revealed the nanoparticles to be predominantly spherical in shape, further confirmed these findings, showing a clear, uniform nanoparticle structure, with an average size range from 35.5 nm to 63.0 nm.

### Safety profile: acute toxicity and LD50 of CO-NC

The safety profile of CO-NC was assessed through acute toxicity studies following oral administration of increasing doses (1000, 3000, 5000, 8000, and 10,000 mg/kg bw) in mice. Notably, no mortality or observable toxic effects were recorded, including histopathological changes such as distorted tissue architecture, congestion, necrosis, or inflammatory cell infiltration. Given these findings, the LD50 of CO-NC was determined to exceed 10,000 mg/kg, highlighting a wide safety margin and confirming its biocompatibility for therapeutic applications.

### Antioxidant capacity of CO-NC

**Fig. 1:**
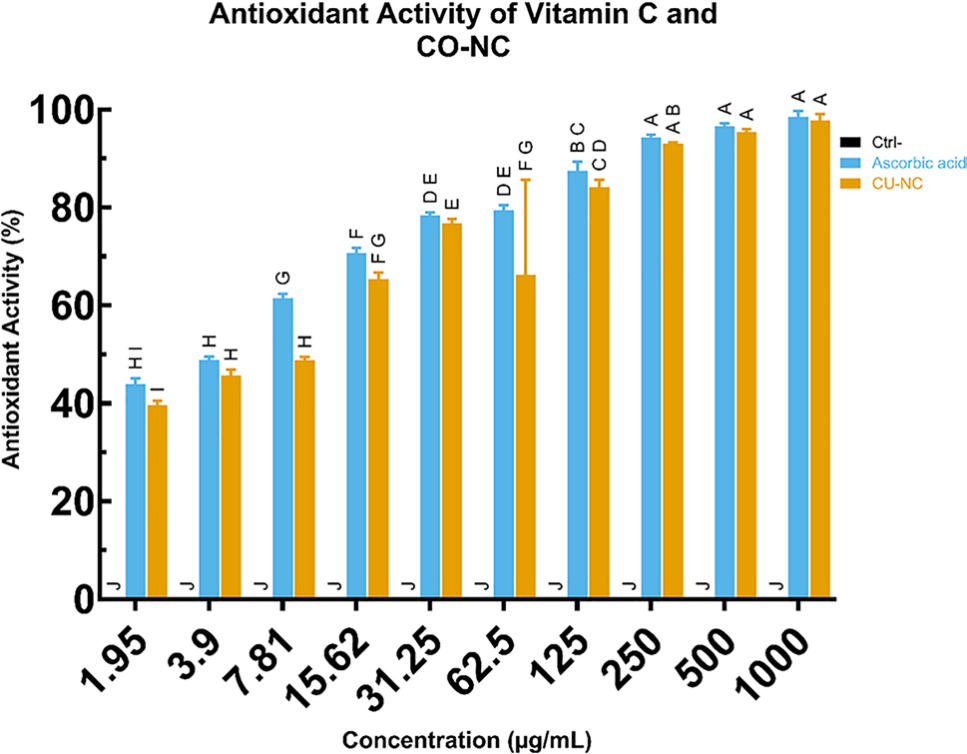
Antioxidant Activity of Vitamin C and CO-NC

Following the confirmation of CO-NC’s safety, its antioxidant activity was evaluated using a DPPH radical scavenging assay. CO-NC demonstrated significant dose-dependent antioxidant activity comparable to ascorbic acid (Fig. 1). At a high concentration (1000 µg/ml), CO-NC achieved a scavenging activity of 97.72%, closely matching that of ascorbic acid (98.5%).

Furthermore, at mid-range concentrations (125 µg/ml), CO-NC retained substantial antioxidant activity (84.2%) relative to ascorbic acid (87.5%), while at lower concentrations (1.95 µg/ml), CO-NC exhibited 39.6% scavenging activity compared to 43.8% for ascorbic acid.

The IC50 values further confirmed the antioxidant potential of CO-NC, with values of 9.28 µg/ml for CO-NC and 5.12 µg/ml for ascorbic acid (Fig. 1D). While CO-NC required slightly higher concentrations to achieve 50% scavenging activity compared to ascorbic acid, these results underscore its robust antioxidant properties and its potential applications in combating oxidative stress.

Antioxidant activity (%) was assessed across varying concentrations of Vitamin C (ascorbic acid) and CO-NC, compared to control (Ctrl-). Both compounds demonstrate increased antioxidant activity with higher concentrations, with CO-NC showing comparable efficacy to Vitamin C at elevated dosages. Data were presented as mean ± SEM, with letters indicating significant differences (*p* < 0.05) determined by ANOVA and Tukey’s post-hoc test.

### Comparative cytotoxicity: CO-NC vs. free curcumin on HepG2 cells

**Fig. 2:**
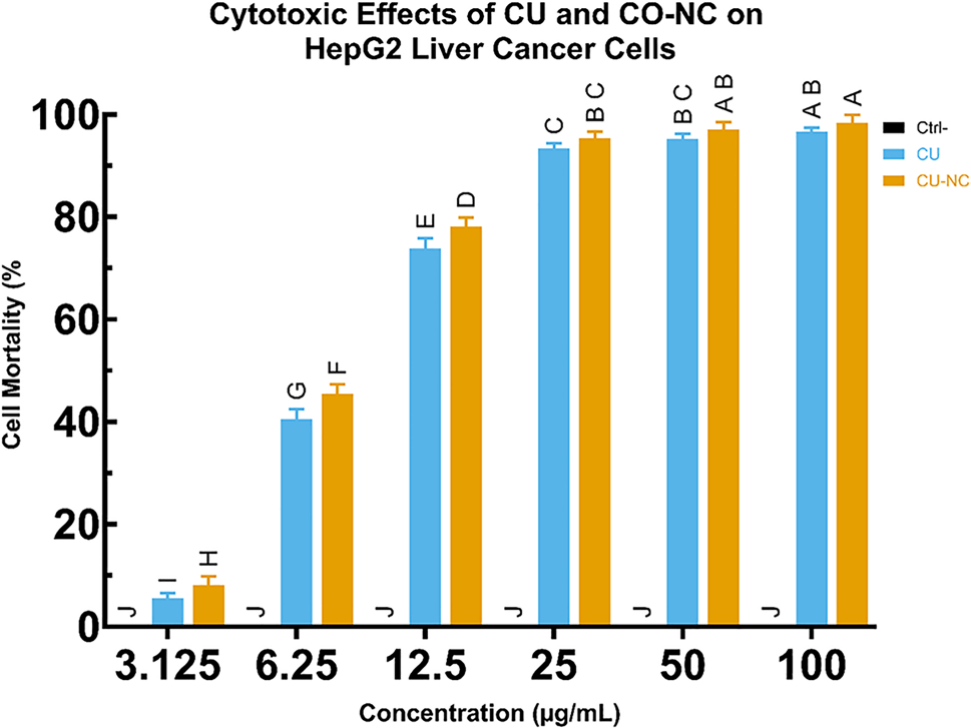
Cytotoxic Effects of CU and CO-NC on HepG2 Liver Cancer Cells

Building upon its antioxidant activity, the cytotoxicity of CO-NC and free curcumin (CU) was evaluated against HepG2 cell lines using the MTT assay (Fig. 2). Both compounds exhibited dose-dependent cytotoxic effects, with CO-NC demonstrating slightly higher cytotoxicity at higher concentrations.

At 100 µg/ml, CO-NC and CU demonstrated cytotoxicity rates of 98.3% and 96.6%, respectively, while at 3.125 µg/ml, cytotoxicity rates significantly decreased to 0.81% (CO-NC) and 0.51% (CU). The calculated IC50 values of 7.62 ± 0.11 µg/ml for CO-NC and 4.62 ± 0.01 µg/ml for CU indicate that CO-NC retains potent anticancer activity. These findings suggest that CO-NC, with its enhanced bioavailability, may serve as a promising anticancer agent.

Cell mortality (%) was measured across varying concentrations of CU and CO-NC, compared to a control group (Ctrl-). Significant increases in cell mortality were observed with higher concentrations of both compounds, with CO-NC demonstrating greater cytotoxicity than CU at higher dosages. Data were presented as mean ± SD, with letters indicating significant differences (*p* < 0.05) determined by ANOVA and post-hoc test.

### Anti-inflammatory properties of CO-NC

**Fig. 3:**
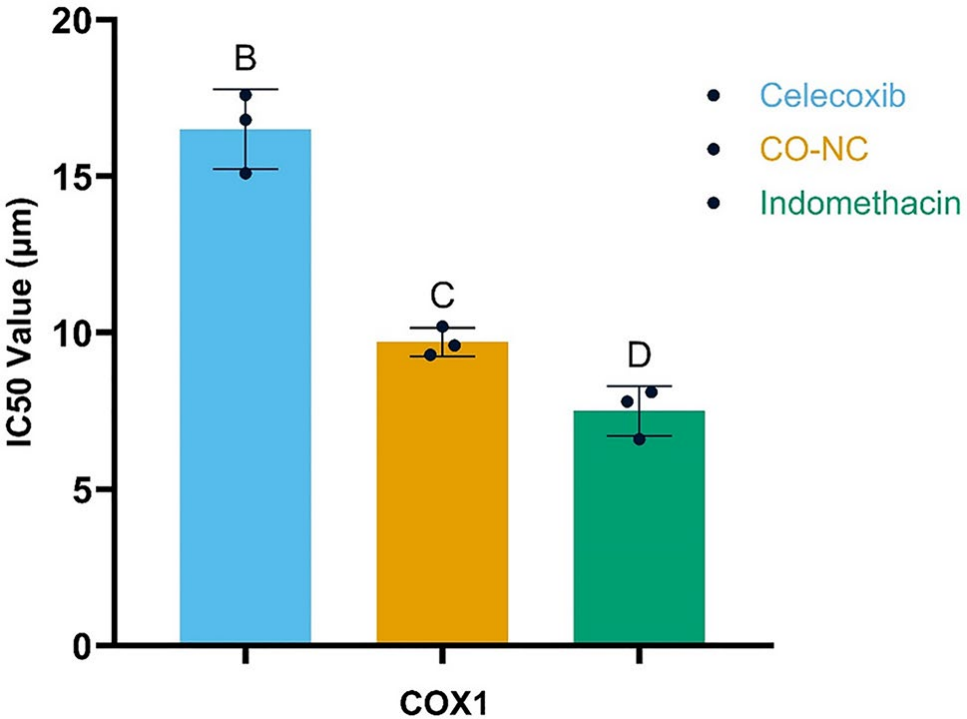
Inhibition of COX1 Activity by CO-NC Compared to Other Compounds

**Fig. 4:**
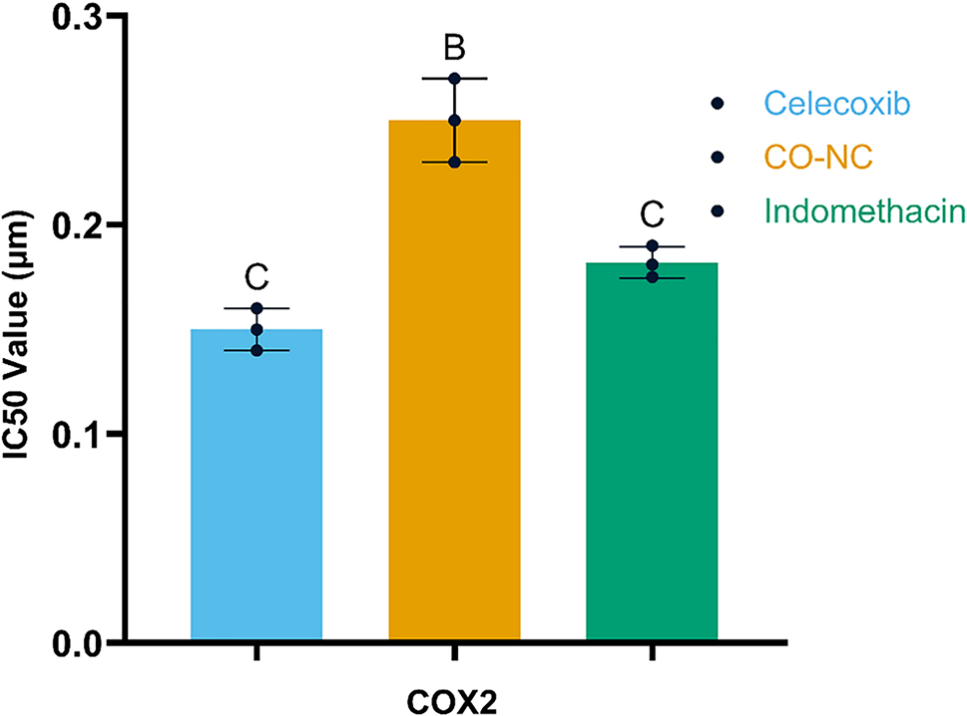
Inhibition of COX2 Activity by CO-NC Compared to Other Compounds

In addition to its cytotoxic effects, the anti-inflammatory potential of CO-NC was assessed by its ability to inhibit COX-1 and COX-2 enzyme activities in vitro (Figs. 3 and 4). CO-NC exhibited a dose-dependent inhibition of both enzymes, with an IC50 value of 9.7 µM for COX-1 and 0.25 µM for COX-2.

Comparatively, standard anti-inflammatory drugs celecoxib and indomethacin showed IC50 values of 16.5 µM and 0.18 µM for COX-1, and 0.15 µM and 0.18 µM for COX-2, respectively. While CO-NC was slightly less potent than the reference drugs, its multifunctional properties and lower toxicity profile underscore its potential as a safer alternative for managing inflammation.

This figure illustrates the inhibitory effects of CO-NC compared to other compounds on COX1 activity, expressed as IC50 (µM). Treatment groups include Celecoxib, CO-NC, and Indomethacin. A significant reduction in COX1 activity was observed with Celecoxib, while CO-NC and Indomethacin demonstrate even greater reductions in activity. Data are presented as mean ± SD. Groups with different letters indicate statistically significant differences (*p* < 0.05), as determined by one-way ANOVA followed by post-hoc tests.

This figure illustrates the inhibitory effects of CO-NC in comparison to other compounds on COX2 activity, expressed as IC50 (µM). Treatment groups include Celecoxib, CO-NC, and Indomethacin. Celecoxib significantly reduces COX2 activity, while CO-NC and Indomethacin demonstrate comparable but slightly lower levels of inhibition. Data are presented as mean ± SD. Groups labeled with different letters indicate statistically significant differences (*p* < 0.05), as determined by one-way ANOVA followed by post-hoc test.

### Trichinocidal activity of CO-NC against adult *T. spiralis*

**Fig. 5:**
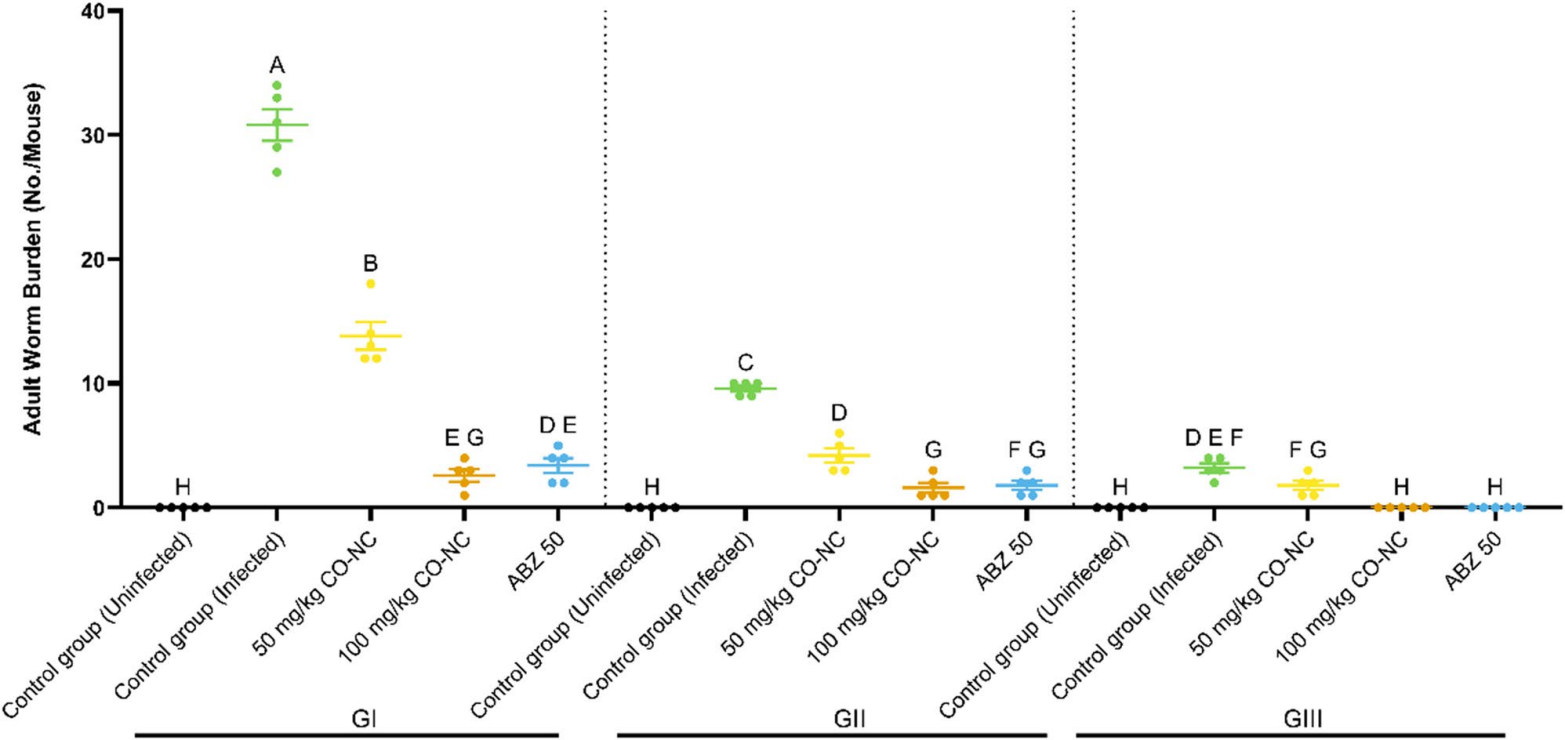
Effectiveness of CO-NC Against *T. spiralis* Adult Worms at Different Concentrations

To assess the anthelmintic efficacy of CO-NC, its ability to reduce adult worm burden in the intestine was compared to ABZ in mice infected with *T. spiralis* (Fig. 5). Treatments administered at 3–5 days post-infection (dpi) resulted in a significant reduction in adult worm counts. Specifically, CO-NC (100 mg/kg) achieved a 91.6% reduction in worm burden at 8 dpi, comparable to ABZ (50 mg/kg), which demonstrated an 88.9% reduction.

Furthermore, at later stages of infection (8–10 dpi), CO-NC achieved an 83.3% reduction in worm burden, similar to ABZ (81.3%). However, during late-stage treatment (33–35 dpi), CO-NC exhibited a 49.3% reduction, slightly higher than ABZ (44.9%). Overall, these results demonstrate that CO-NC was highly effective in targeting intestinal worms, particularly at early stages of infection.

This figure illustrates the adult worm burden (number of adult worms per mouse) across different treatment groups, including control groups (infected and uninfected), two concentrations of CO-NC (50 and 100 mg/kg), and ABZ (50 mg/kg), categorized into three groups: Group I (GI), treated on days 3, 4, and 5 post-infection (dpi); Group II (GII), treated on days 8, 9, and 10 dpi; and Group III (GIII), treated on days 33, 34, and 35 dpi. Data are presented as mean ± SEM. Groups with different letters indicate statistically significant differences (*p* < 0.05), as determined by one-way ANOVA followed by post-hoc tests. CO-NC, compound; ABZ, albendazole.

### Efficacy of CO-NC on Tissue-Dwelling *T. spiralis* larvae

**Fig. 6:**
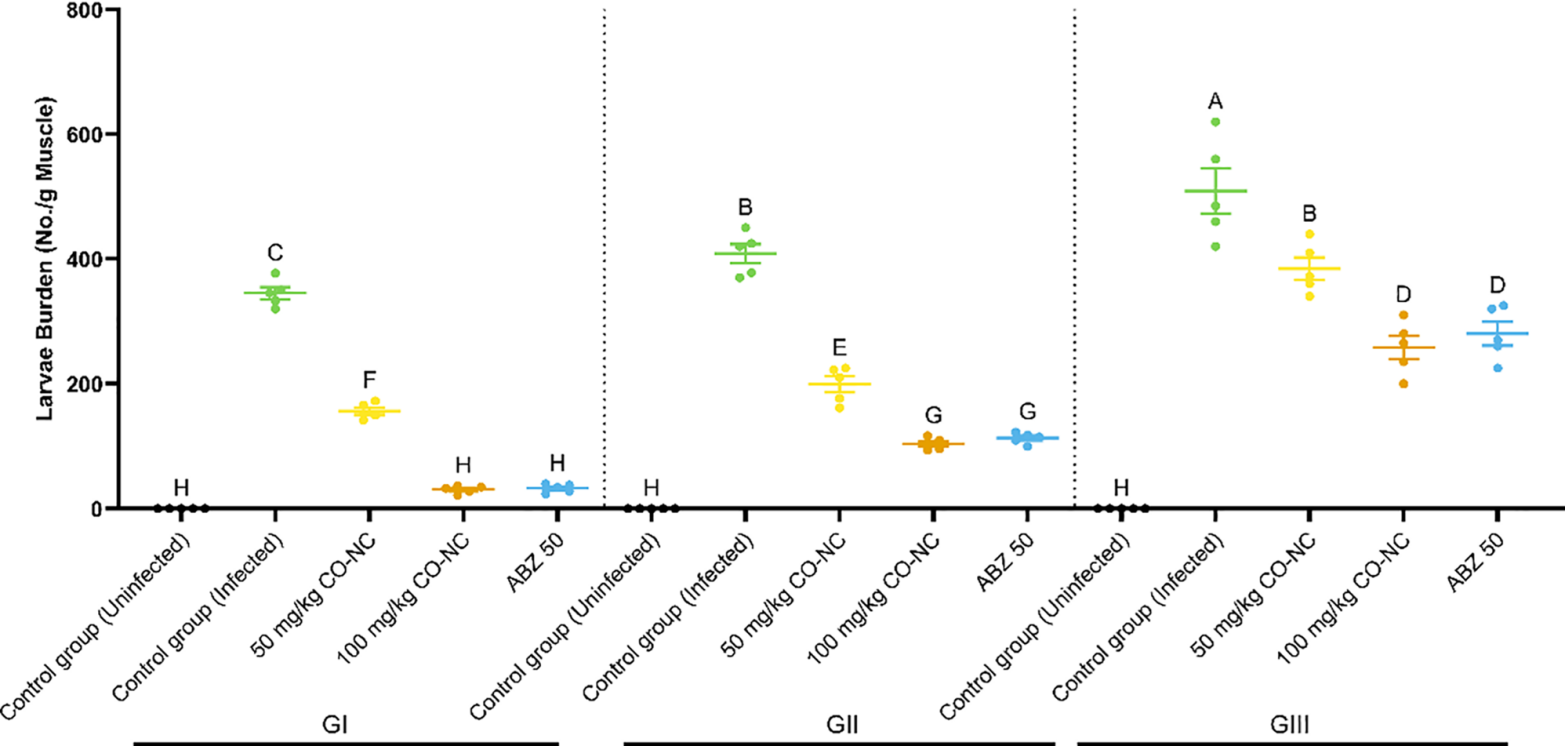
Effectiveness of CO-NC Against *T. spiralis* Larvae at Different Concentrations

In addition to its efficacy against adult worms, the ability of CO-NC to reduce encapsulated *T. spiralis* larvae in muscle tissues was assessed (Fig. 6). Early treatment (3–5 dpi) with CO-NC (100 mg/kg) significantly reduced the number of larvae by 91%, comparable to ABZ (90.7%).

However, at 8–10 dpi, the efficacy of CO-NC decreased to 74.5% for 100 mg/kg, while ABZ showed a similar efficacy of 72.3%. At later stages of infection (33 dpi), the efficacy of both CO-NC and ABZ decreased further, with reductions of 49.3% and 44.9%, respectively. These findings suggest that early intervention was critical for maximizing the therapeutic potential of CO-NC.

The figure depicts the larval burden (number of larvae per gram of muscle) across various treatment groups, including control groups (infected and uninfected), two concentrations of CO-NC (50 and 100 mg/kg), and ABZ (50 mg/kg) in Group I (GI), treated on days 3, 4, and 5 post-infection (dpi); Group II (GII), treated on days 8, 9, and 10 dpi; and Group III (GIII), treated on days 33, 34, and 35 dpi. Data are expressed as mean ± SEM. Groups labeled with different letters indicate statistically significant differences (*p* < 0.05), as determined by one-way ANOVA followed by post-hoc tests. CO-NC, compound; ABZ, albendazole.

### Impact of CO-NC treatment on host redox parameters

**Fig. 7:**
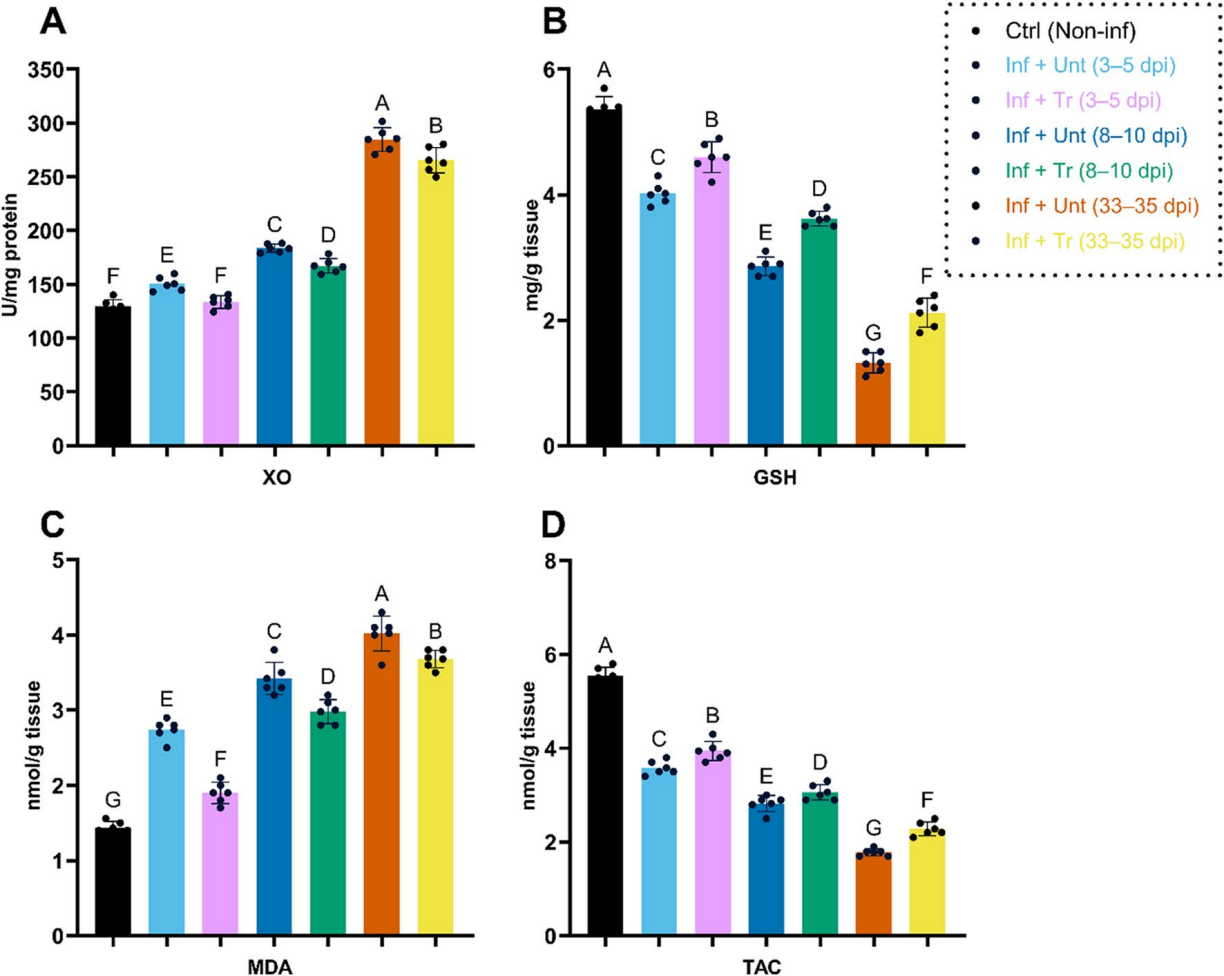
Redox Parameters in Small Intestinal Homogenates of *T. spiralis*-Infected and Treated Mice

**Fig. 8:**
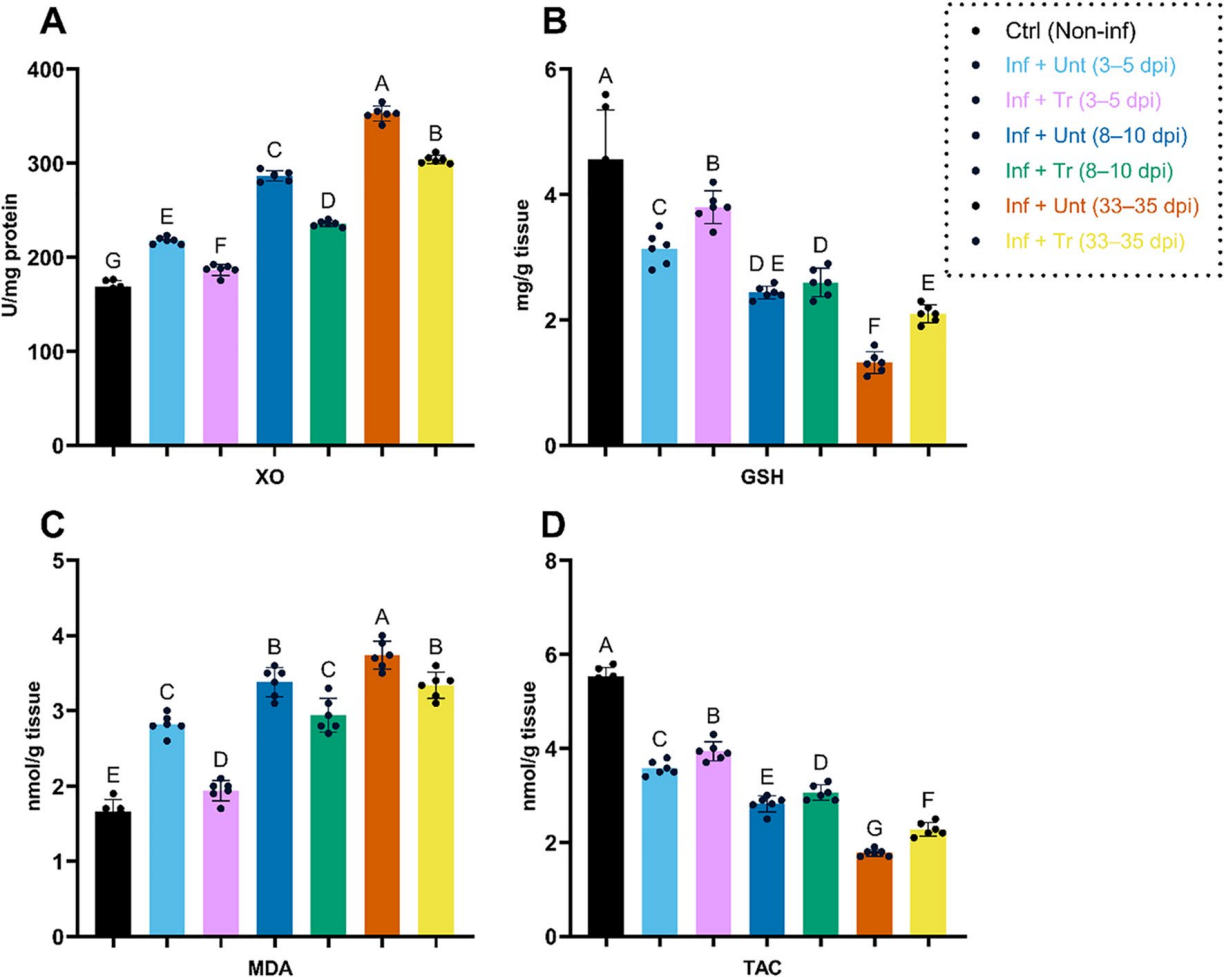
Redox Parameters in Skeletal Muscle Homogenates of *T. spiralis*-Infected and Treated Mice

Finally, the effects of CO-NC on oxidative stress markers and antioxidant levels in small intestinal and skeletal muscle homogenates of infected mice were evaluated (Figs. 7 and 8). Infection with *T. spiralis* induced significant oxidative stress in both tissues, as evidenced by increased XO activity and MDA levels, along with GSH levels and TAC. Early treatment (3–5 dpi) with CO-NC (100 mg/kg) significantly decreased oxidative stress markers, such as MDA and XO, while increasing antioxidant markers, including GSH and TAC.

At later stages of infection (8–10 dpi and 33 dpi), the efficacy of CO-NC in restoring redox balance remained statistically significant compared to untreated controls. These findings highlight the importance of therapeutic interventions, particularly early treatment, in mitigating oxidative stress and improving tissue health during parasitic infections.

This figure illustrates the changes in key redox parameters in small intestinal homogenates across different experimental groups. Measurements include:

(A) Xanthine oxidase (XO) activity, (B) Glutathione (GSH) levels, (C) Malondialdehyde (MDA) concentrations, and (D) Total antioxidant capacity (TAC). Data were presented as mean ± SD. Groups marked with different letters indicate statistically significant differences (*p* < 0.05) as determined by one-way ANOVA followed by post-hoc tests. The dotted box highlights the control group (Ctrl, Non-inf) and treatment time points. dpi, days post-infection; Inf, infected; Tr, treated; Unt, untreated.

This figure illustrates the changes in key redox parameters in skeletal muscle homogenates across different experimental groups. Measurements include:

(A) Xanthine oxidase (XO) activity, (B) Glutathione (GSH) levels, (C) Malondialdehyde (MDA) concentrations, and (D) Total antioxidant capacity (TAC).Data were presented as mean ± SD. Groups marked with different letters indicate statistically significant differences (*p* < 0.05) as determined by one-way ANOVA followed by post-hoc tests. The dotted box highlights the control group (Ctrl, Non-inf) and treatment time points.

## Discussion

### Overview of CO-NC formulation and properties

This study evaluated the efficacy of CO-NC as a potential treatment for *T. spiralis* infection, a zoonotic nematode that causes trichinellosis and poses a significant public health risk worldwide [33]. Current treatment options for trichinellosis are limited, particularly in eradicating encysted or newly migrating larvae, and there is growing concern about drug resistance [34]. Our findings demonstrated that CO-NC, a nanoformulated version of curcumin, significantly enhanced the therapeutic efficacy of curcumin by overcoming its intrinsic limitations, such as poor solubility, bioavailability, and limited organ penetration [35, 36]. The advantages of nanoformulation, including enhanced solubility, bioavailability, and cell-penetrating ability, were particularly evident in our results.

The study focused on assessing the anti-inflammatory, antioxidant, and trichinocidal properties of CO-NC, alongside its safety profile, when used to target different stages of *T. spiralis* infection in mice. Compared to crude curcumin and conventional reference drugs, CO-NC exhibited superior efficacy, particularly in mitigating oxidative stress, reducing inflammation, and eradicating the parasite. It is important to note that while the present study demonstrated the promising antiparasitic and antioxidant potential of CO-NC, the stability of this nanocomposite under different environmental factors, such as temperature, humidity, and light exposure, was not investigated. Future studies are highly recommended to evaluate the long-term stability of CO-NC under such conditions, as this will be critical for its potential clinical and commercial applications. Specifically, the study underscored the critical role of oxidative stress and inflammation in *T. spiralis* pathology, as these processes exacerbate tissue damage and parasite survival. CO-NC directly targeted these mechanisms, highlighting its therapeutic potential. These findings not only confirm the potential of CO-NC as an antiparasitic agent but also underscore the broader advantages of nanoformulations in enhancing drug performance.

### Safety and radical scavenging activity

Our results demonstrated that CO-NC has an excellent safety profile, as no toxicity symptoms were observed in mice even at doses as high as 10,000 mg/kg bw. The LD50 exceeded the highest tested dose, indicating a wide safety margin. This finding is particularly significant, as safety concerns often limit the clinical application of many natural compounds.

In terms of antioxidant activity, CO-NC exhibited significantly higher radical scavenging activity compared to Vitamin C, a well-established antioxidant. This dose-dependent activity was likely due to the enhanced phenolic content of the nanoformulation, which surpasses that of crude curcumin [11]. The antioxidant activity of CO-NC also mitigated oxidative stress caused by *T. spiralis*, as evidenced by decreased levels of XO and MDA and increased levels of GSH and TAC in treated mice. By reducing reactive oxygen species (ROS) production, CO-NC directly addressed a key pathological feature of *T. spiralis* infection.

### Anti-inflammatory effects and COX inhibition

One of the key findings of this study was the potent anti-inflammatory activity of CO-NC. The nanoformulation effectively inhibited COX1 and COX2 enzymes at lower doses than crude curcumin, highlighting its enhanced solubility and cell-penetrating capabilities. Given that inflammation in *T. spiralis* infection is characterized by elevated COX2 immunoreactivity, which exacerbates oxidative stress [37], the ability of CO-NC to reduce COX2 production represents a critical therapeutic advantage.This reduction in COX2 activity was associated with decreased apoptosis and reduced angiogenesis, two processes that contribute to tissue damage and parasite survival during *T. spiralis* infection.

Furthermore, CO-NC’s inhibition of prostaglandin production was comparable to that of indomethacin, a widely used anti-inflammatory drug, and superior to celecoxib. By selectively targeting COX2, CO-NC effectively reduced inflammation while minimizing potential gastrointestinal toxicity, a significant advantage over traditional COX inhibitors [38]. The importance of COX2 inhibition in parasite clearance and host recovery [39] further highlights the therapeutic potential of CO-NC, aligning with previous studies.

The anti-inflammatory role of CO-NC played an important role in mitigating tissue damage caused by the parasite. This effect results from affecting eicosanoid signaling pathway with transcriptional and post-transcriptional regulation of cyclooxygenase and lipoxygenase enzymes [14, 40]. COX-2 showed increased intestinal and tissue levels during trichinellosis infection with hypermotility and interference with muscle regeneration [41, 42].

### Efficacy against different stages of *T. spiralis*

CO-NC demonstrated remarkable trichinocidal efficacy against all stages of *T. spiralis*, particularly when administered at critical time points post-infection. The nanoformulation effectively eradicated adult worms in the intestine when administered at 8–10 dpi and killed newly migrating larvae when administered between 3 and 8 dpi. This timing aligns with the parasite’s life cycle, as *T. spiralis* completes migration and cyst development in mice by 12–14 dpi. The disappearance of intestinal worms by 33 dpi was attributed to both the natural life cycle of the parasite, and its natural life span in the gut, and the therapeutic action of CO-NC [6, 43]. Of particular importance was the ability of CO-NC to reduce the number of encapsulated larvae in muscle tissue at 33 dpi, from 509 ± 80.3 larvae per gram in the control group to 258 ± 42.2 larvae per gram in the treated group. This was accompanied by a reduction in the infectivity of larvae in new host mice, suggesting that CO-NC could penetrate the cyst wall and exert its effects on late-encysted larvae. These findings align with prior studies on natural compounds such as resveratrol, which similarly demonstrated efficacy against *T. spiralis* [10]. Curcumin anti-parasitic efficacy is multifactorial including inflammatory response amelioration, improving muscle cells regeneration, angiogenetic effect and interfering with nurse cells formation. Nurse cell formation is crucial for survival of the larvae as it essential for nutrition and waste elimination [44].

The declined efficacy of the CO-NC when administered later (33 dpi) could be attributed to the formation of the tissue cyst surrounding the larvae that forms a barrier against drugs penetration, however, it was still superior to the reference drug. On the other hand, curcumin is known by its rapid metabolism, however, the nanoformulation improved its bioavailability [45]. The ability of CO-NC to infiltrate host cells and reach larvae before cyst wall formation is particularly noteworthy. This property may explain its enhanced efficacy compared to reference drugs and crude curcumin [46].

### Antioxidant and redox-modulating effects

Oxidative stress, induced by excessive production of ROS during *T. spiralis* infection, plays a critical role in parasite survival and host pathology [23]. CO-NC exhibited robust antioxidant activity, as evidenced by decreased levels of XO and MDA and increased levels of GSH and TAC in treated mice. These effects were particularly pronounced at earlier post-infection intervals (3–5 dpi and 8–10 dpi), consistent with previous findings [10, 18].

The enhanced antioxidant capacity of CO-NC compared to crude curcumin further supports the hypothesis that nanoformulation improves pharmacological properties, such as organ penetration and bioavailability [44]. Overall, these findings reinforce the importance of antioxidant-rich therapies in mitigating parasite-induced oxidative damage and enhancing drug efficacy [37].

### Comparative efficacy with reference drugs

When compared to albendazole, a standard reference drug for trichinellosis, CO-NC (100 mg/kg bw) demonstrated comparable efficacy, effectively eradicating adult worms and newly migrating larvae. However, CO-NC offered additional benefits, such as its antioxidant and anti-inflammatory properties, which contributed to improved host recovery and reduced oxidative stress. Moreover, CO-NC was able to penetrate the cyst wall and target encysted larvae, a feature not observed in ABZ. These findings reinforce CO-NC’s multifunctional advantages over conventional therapies [47]. Plant-derived nanocomposite represents a promising natural product with broad therapeutic potential in parasitic disease management [48].

### Limitations and future directions

Despite its promising results, this study has limitations that warrant further investigation. First, the use of a mouse model restricts direct extrapolation to human applications, necessitating additional studies in larger animal models and clinical trials. Second, the stability of CO-NC under physiological conditions, such as varying gastric and intestinal pH environments, was not evaluated in this study. Assessing the nanocomposite’s behavior in simulated gastrointestinal fluids will be critical for understanding its oral bioavailability and therapeutic efficacy in vivo. Third, the long-term stability of CO-NC under different environmental factors such as temperature, humidity, and light exposure remains to be addressed.

Moreover, scalability of CO-NC production for mass manufacturing requires further optimization. While this study focused on laboratory-scale formulation, future work should investigate scalable, reproducible manufacturing processes that maintain product consistency and quality. Consideration of cost-effectiveness and industrial feasibility will be essential for clinical translation and commercial application. Advanced production methods may increase manufacturing costs, which can impact the feasibility of widespread use. Furthermore, individual testing of materials such as Tween 80, olive oil, and the nano-formulation was not performed in this study.

Future research should focus on optimizing the CO-NC formulation for clinical applications by refining the dose and treatment duration, especially to ensure complete eradication of encapsulated larvae. Additionally, exploring its pharmacokinetics, biodistribution, and potential synergistic effects with existing therapeutic agents will provide critical insights into its suitability for human use. Expanding this research to assess CO-NC’s efficacy against other parasitic infections can further validate its versatility and therapeutic potential.

## Conclusion

This study demonstrates that CO-NC exhibits significant anthelmintic efficacy against *T. spiralis*, effectively targeting adult worms, reducing newborn larvae, and minimizing encapsulated larvae in muscle tissues. The nanoformulation offers several key advantages, including enhanced bioavailability, potent anti-inflammatory and antioxidant properties, a wide safety margin, and an eco-friendly profile. These features position CO-NC as a multifunctional therapeutic agent that outperforms conventional treatments.

While further optimization is required to address certain limitations, such as scalability and dose refinement, the findings of this study underscore the transformative potential of CO-NC in parasitic disease treatment. Beyond *T. spiralis*, CO-NC’s nanoformulation approach paves the way for broader applications in other parasitic diseases and related therapeutic contexts, including cancer and inflammation-driven disorders.

Ultimately, CO-NC represents a promising, innovative, and sustainable solution to address the challenges of parasitic infections. Future research should continue to build upon this foundation, exploring its integration into existing therapeutic protocols and its potential for advancing the field of nanotechnology-based medicine.

Taken together, these results demonstrate that CO-NC was a safe, multifunctional therapeutic agent with significant antioxidant, anti-inflammatory, and anthelmintic efficacy. Its ability to improve redox parameters, reduce worm burden, and minimize tissue damage highlights its potential as a novel treatment for parasitic infections.

## Data Availability

Data is provided within the manuscript.

## Abbreviations

dpi: days post-infection
Inf: infected
Tr: treated
Unt: untreated

## References

[1] Abuelenain GL, Fahmy ZH, Elshennawy AM, Fahmy AM, Ali EM, Hammam O, Abdel-Aziz AWA. The potency of Lepidium sativum and Commiphora molmol extracts on Trichinella spiralis stages and host interaction. BMC Vet Res. 2021;9(9):1376–82.

[2] Abdel-Hakeem SS, Abdel-Samiee MAZ, Youssef MSE, Abd-Elsadek SH, Abd-Elrahman SM, Abdel-Hakeem SS. Nanocurcumin: a promising therapeutic candidate for experimental trichinellosis. Microsc Microanal. 2024;30:368–81. 38323506 10.1093/micmic/ozae007

[3] Ali MF, Sadek AS, Abd Elghfar SK, Taha M. Nano-curcumin attenuates nephropathic lesions induced by chronic ketoprofen administration in rats: role of cyclooxygenase-1. J Adv Veterinary Res. 2022;12:524–34.

[4] Atia AF, Abokhalil NA, Sweed DM, Moaz IM, Abou Hussien NM. Curcumin nanoparticles versus Curcumin in amelioration of inflammatory and pathological changes during the migratory phase of murine trichinellosis. J Egypt Soc Parasitol. 2021;51:239–56.

[5] Abd El-Hamed W, Abd-Rabou A, Faramawy A. Therapeutic efficacy of curcuma and pomelo loaded Chitosan nanoparticles in intestinal murine trichinellosis. Egypt J Chem. 2022;65(2):551–64.

[6] Chinedu E, Arome D, Ameh FS. A new method for determining acute toxicity in animal models. Toxicol Int. 2013;20:224–6. 24403732 10.4103/0971-6580.121674 PMC3877490

[7] Rai M, Ingle A, Pandit R, Paralikar P, Anasane N, Santos C. Curcumin and curcumin-loaded nanoparticles: antipathogenic and antiparasitic activities. Expert Rev Anti-Infective Therapy. 2020;18:367–79. 32067524 10.1080/14787210.2020.1730815

[8] Abo-Hussien N, Soliman S, Sweed D, Mandour S, Abokhalil N, Atia A. Evaluation of the therapeutic efficacy of Curcumin nanoparticles in the intestinal and muscular phases of murine trichinosis. Parasitologists United J. 2023;16:230–45.

[9] El-Wakil ES, Abdelmaksoud HF, AbouShousha TS, Ghallab MMI. Evaluation of Annona muricata (Graviola) leaves activity against experimental trichinellosis: in vitro and in vivo studies. J Helminthol. 2021;95:e53. 34515021 10.1017/S0022149X21000481

[10] Elgendy DI, Othman AA, Hasby Saad MA, Soliman NA, Mwafy SE. Resveratrol reduces oxidative damage and inflammation in mice infected with Trichinella spiralis. J Helminthol. 2020;94:e140. 32238206 10.1017/S0022149X20000206

[11] Khalifa MM, Ramadan RM, Youssef FS, Auda HM, El-Bahy MM, Taha NM. Trichinocidal activity of a novel formulation of curcumin-olive oil nanocomposite in vitro. Veterinary Parasitology: Reg Stud Rep. 2023;41:100880. 10.1016/j.vprsr.2023.100880 37208086

[12] Jeevanandam J, San Chan Y, Danquah MK. Nano-formulations of drugs: recent developments, impact and challenges. Biochimie. 2016;128:99–112. 27436182 10.1016/j.biochi.2016.07.008

[13] Alsuraihi A, Salam MA, Elbialy N. Physicochemical properties of ZnONPs synthesized by chemical, sonochemical, and green methods: comparative study. Appl Phys A. 2023;129(11):768.

[14] Hamed AMR, Abdel-Shafi IR, Elsayed MDA, Mahfoz AM, Tawfeek SE, Abdeltawab MSA. Investigation of the effect of Curcumin on oxidative stress, local inflammatory response, COX-2 expression, and microvessel density in Trichinella spiralis-induced enteritis, myositis and myocarditis in mice. Helminthologia. 2022;59:18–36. 35601760 10.2478/helm-2022-0002 PMC9075878

[15] Taha NM, Youssef FS, Auda HM, El-Bahy MM, Ramadan RM. Efficacy of silver nanoparticles against Trichinella spiralis in mice and the role of multivitamin in alleviating its toxicity. Sci Rep. 2024;14(1):5843. 38462650 10.1038/s41598-024-56337-2 PMC10925591

[16] Taha NM, Salem MA, El-Saied MA, Mohammed FF, Kamel M, El-Bahy MM, Ramadan RM. Multifaceted analysis of equine cystic echinococcosis: genotyping, immunopathology, and screening of repurposed drugs against E. equinus protoscolices. BMC Vet Res. 2025;21(1):178. 40098107 10.1186/s12917-025-04616-z PMC11912610

[17] Kandeel M, Ur Rehman T, Akhtar T, Zaheer T, Omar M. Anti-parasitic applications of nanoparticles: a review. Pakistan Veterinary J. 2022;42:2074–7764.

[18] Kazemzadeh H, Mohammad F. Evaluating expression of oxidative stress genes in response to Trichinella spiralis infection. Indian J Sci Res. 2014;5:305–9.

[19] Moryani AA, Rajput N, Naeem M, Hussain A, Jahejo AR. Screening of the herbs and evaluation of their combined effects on the health and immunity of coccidiosis-challenged broiler chickens. Pakistan Veterinary J. 2021;41:2074–7764.

[20] Mayer-Scholl A, Pozio E, Gayda J, Thaben N, Bahn P, Nöckler K. Magnetic stirrer method for the detection of *Trichinella* larvae in muscle samples. J Visualized Experiments. 2017;(121):55354. 10.3791/55354 PMC5409328 28287594

[21] Ramadan RM, Salem MA, Mohamed HI, Orabi A, El-Bahy MM, Taha NM. Dermanyssus gallinae as a pathogen vector: phylogenetic analysis and associated health risks in pigeons. Vet Parasitol Reg Stud Rep. 2025;57:101198. 10.1016/j.vprsr.2025.101198 39855842

[22] Salem MA, Taha NM, El-Bahy MM, Ramadan RM. Phylogenetic position of the pigeon mite, ornithonyssus Sylviarum, with amplification of its Immunogenetic biomarkers in Egypt. Sci Rep. 2024;14(1):22026. 39322649 10.1038/s41598-024-72433-9 PMC11424627

[23] Othman AA, Abou Rayia DM, Ashour DS, Saied EM, Zineldeen DH, El-Ebiary AA. Atorvastatin and Metformin administration modulates experimental Trichinella spiralis infection. Parasitol Int. 2016;65:105–12. 26546571 10.1016/j.parint.2015.11.001

[24] Ramadan RM, Wahby AM, Bakry NM, Auda HM, Mohammed FF, El-Bahy MM, Abdalla Hekal SH. Targeted pre-partum strategies to suppress Toxocara vitulorum hypobiotic larvae: reducing transmission to calves and genotypic insights into Buffalo infections. Vet World. 2025;18(2). 10.14202/vetworld.2025.329-340 PMC11963570 40182831

[25] Jacob S, Nair AB. An updated overview on therapeutic drug monitoring of recent antiepileptic drugs. Drugs R D. 2016;16:303–16. 27766590 10.1007/s40268-016-0148-6 PMC5114206

[26] Taha NM, Zalat RS, Khaled E, Elmansory BM. Evaluation of the therapeutic efficacy of some essential oils in experimentally immunosuppressed mice infected with Cryptosporidium parvum. J Parasit Dis. 2023;47(4):733–43. 38009149 10.1007/s12639-023-01621-7 PMC10667177

[27] Khalifa MM, Salem MA, Fouad EA, Bakry NM, Kamel MS, El-Bahy MM, Ramadan RM. Vector-borne pathogens in dogs in Egypt: molecular and immunological insights. Res Vet Sci. 2025;105629. 10.1016/j.rvsc.2025.105629 40157237

[28] Ramadan RM, Bakr AF, Fouad E, Mohammed FF, Abdel-Wahab AM, Abdel-Maogood SZ, El-Bahy MM, Salem MA. Novel insights into antioxidant status, gene expression, and immunohistochemistry in an animal model infected with camel-derived trypanosoma evansi and theileria annulata. Parasites Vectors. 2024;17(1):474. 39558410 10.1186/s13071-024-06564-3 PMC11575088

[29] Khalifa MM, Mohamed HI, Ramadan RM, Youssef FS, El-Bahy MM, Abdel-Radi S. Smart application of silver nanoparticles in the treatment of chicken coccidiosis in combination with special supplement to alleviate its toxicity. Vet Parasitol. 2025;110440. 10.1016/j.vetpar.2025.110440 40054330

[30] Ramadan RM, Taha NM, Auda HM, Elsamman EM, El-Bahy MM, Salem MA. Molecular and immunological studies on theileria equi and its vector in Egypt. Exp Appl Acarol. 2024;93(2):439–58. 38967736 10.1007/s10493-024-00933-4 PMC11269342

[31] Taha NM, Sabry MA, El-Bahy MM, Ramadan RM. Awareness of parasitic zoonotic diseases among pet owners in Cairo, Egypt. Vet Parasitol Reg Stud Rep. 2024;51:101025. 10.1016/j.vprsr.2024.101025 38772640

[32] Mahdy OA, Ramadan RM, Salem MA. Innovative molecular and immunological approaches of heterophyiasis infecting some Egyptian marketed fishes. BMC Vet Res. 2024;20(1):385. 39215340 10.1186/s12917-024-04226-1 PMC11363687

[33] Anisuzzaman, Hossain MS, Hatta T, Labony SS, Kwofie KD, Kawada H, et al. Food- and vector-borne parasitic zoonoses: global burden and impacts. Adv Parasitol. 2023;120:87–136. 36948728 10.1016/bs.apar.2023.02.001

[34] Taher EE, Méabed EMH, El Akkad DMH, Kamel NO, Sabry MA. Modified dot-ELISA for diagnosis of human trichinellosis. Exp Parasitol. 2017;177:40–6. 28438521 10.1016/j.exppara.2017.04.002

[35] Sarwar I, Ashar A, Mahfooz A, Aqib AI, Kulyar MF. Evaluation of antibacterial potential of Raw turmeric, nano-turmeric, and NSAIDs against multiple drug resistant Staphylococcus aureus and E. coli isolated from animal wounds. Pak Vet J. 2021;41:209–14.

[36] Gera M, Sharma N, Ghosh M, Huynh DL, Lee SJ, Min T, et al. Nanoformulations of Curcumin: an emerging paradigm for improved remedial application. Oncotarget. 2017;8(39):66680. 29029547 10.18632/oncotarget.19164 PMC5630447

[37] Abdeltawab MS, Abdel-Shafi IR, Aboulhoda BE, Mahfoz AM, Hamed A. The neuroprotective potential of Curcumin on T. spiralis infected mice. BMC Complement Med Ther. 2024;24:99. 38388410 10.1186/s12906-024-04399-0 PMC10882799

[38] Unsal-Tan O, Ozadali K, Piskin K, Balkan A. Molecular modeling, synthesis and screening of some new 4-thiazolidinone derivatives with promising selective COX-2 inhibitory activity. Eur J Med Chem. 2012;57:59–64. 23047224 10.1016/j.ejmech.2012.08.046

[39] Turkmen R, Birdane YO, Demirel HH, Kabu M, Ince S. Protective effects of Resveratrol on biomarkers of oxidative stress, biochemical and histopathological changes induced by sub-chronic oral glyphosate-based herbicide in rats. Toxicol Res. 2019;8:238–45. 10.1039/c8tx00287h PMC6417488 30997023

[40] Rao CV. Regulation of COX and LOX by Curcumin. Adv Exp Med Biol. 2007;595:213–26. 17569213 10.1007/978-0-387-46401-5_9

[41] Barbara G, De Giorgio R, Deng Y, Vallance B, Blennerhassett P, Collins SM. Role of Immunologic factors and cyclooxygenase 2 in persistent postinfective enteric muscle dysfunction in mice. Gastroenterology. 2001;120(7):1729–36. 11375954 10.1053/gast.2001.24847

[42] El-Aswad BEW, Amar AI, Mahmoud SF, Soliman SS, Abd El-Atty AF. Immunohistochemical evaluation of interleukin-23 and cyclooxygenase-2 in the muscles of mice infected with Trichinella spiralis. Trop Biomed. 2020;37(1):75–88. 33612720

[43] Takahashi Y. Biology of Trichinella. Trichinella and trichinellosis. Elsevier; 2021. pp. 77–101.

[44] Khedr SI, Gomaa MM, Mogahed NMFH, Gamea GA, Khodear GAM, Sheta E, et al. Trichinella spiralis: A new parasitic target for Curcumin nanoformulas in mice models. Parasitol Int. 2024;98:102810. 37730195 10.1016/j.parint.2023.102810

[45] Anand P, Kunnumakkara AB, Newman RA, Aggarwal BB. Bioavailability of Curcumin: problems and promises. Mol Pharm. 2007;4(6):807–18. 17999464 10.1021/mp700113r

[46] Atia A, El-Kersh W, El-Nahas N, Moharm I, Lasheen M, Abo-Hussien N. Therapeutic efficacy of Trichinella spiralis nano-cathepsin B antigen in murine trichinosis. Parasitologists United J. 2023;16:208–19.

[47] Yadav AK. Temjenmongla. Efficacy of Lasia spinosa leaf extract in treating mice infected with Trichinella spiralis. Parasitol Res. 2012;110:493–8. 21748345 10.1007/s00436-011-2551-9

[48] Bakr AF, Youssef FS, Bahr AD, Ramadan RM. Health benefits of Astragalus polysaccharides and possible techniques for upgrading their efficiency: A comprehensive review. Egypt J Chem. 2024;67(11):29–44.

